# Retinal sublayer defect is independently associated with the severity of hypertensive white matter hyperintensity

**DOI:** 10.1002/brb3.1521

**Published:** 2019-12-25

**Authors:** Man Qu, William Robert Kwapong, Chenlei Peng, Yungang Cao, Fan Lu, Meixiao Shen, Zhao Han

**Affiliations:** ^1^ The Second Affiliated Hospital and Yuying Children's Hospital of Wenzhou Medical University Wenzhou China; ^2^ Taizhou Central Hospital Taizhou University Hospital Taizhou China; ^3^ School of Ophthalmology and Optometry Wenzhou Medical University Wenzhou China

**Keywords:** Fazekas scale, retina, spectral‐domain optical coherence tomography, white matter hyperintensity

## Abstract

**Purpose:**

To investigate the association of specific retinal sublayer thicknesses on optical coherence tomography (OCT) imaging with brain magnetic resonance imaging (MRI) markers using the Fazekas scale in hypertensive white matter hyperintensity (WMH) subjects.

**Methods:**

Eighty‐eight participants (32 healthy controls and 56 hypertensive white matter hyperintensity subjects) underwent retinal imaging using the OCT and MRI. A custom‐built algorithm was used to measure the thicknesses of the retinal nerve fiber layer (RNFL) and ganglion cell layer and inner plexiform layer (GCIP). Focal markers for white matter hyperintensities were assessed on MRI and graded using the Fazekas visual rating.

**Results:**

Hypertensive WMH showed significantly reduced (*p* < .05) RNFL and GCIP layers when compared to healthy controls, respectively. A significant correlation was found between the RNFL (*ρ* = −.246, *p* < .001) and GCIP (*ρ* = −.338, *p* < .001) of the total participants and the Fazekas score, respectively. Statistical differences were still significant (*p* < .05) when correlations were adjusted for intereye correlation, age, hypertension, smoking, body mass index, and diabetes. Among the cases of hypertensive WMH, higher Fazekas scores were significantly associated (*p* < .05) with the thinning of both the RNFL and GCIP layers after adjustment of age and other risk factors.

**Conclusions:**

Retinal degeneration in the RNFL and GCIP was independently associated with focal lesions in the white matter of the brain and deteriorates with the severity of the lesions. We suggest that imaging and measurement of the retinal sublayers using the OCT may provide evidence on neurodegeneration in WMH.

## Graphical abstract

In our current study, we conducted an observational study to investigate the association of the retinal sublayer thickness on OCT with focal markers of brain tissue on MRI in participants with WMH using the Fazekas scale. Retinal degeneration in the RNFL and GCIP was independently associated with focal lesions in the white matter of the brain and deteriorates with the severity of the lesions. We suggest that imaging and measurement of the retinal sublayers using the OCT may provide evidence on neurodegeneration in WMH.

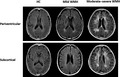

## 1 INTRODUCTION

White matter hyperintensities (WMH), a typical indication of cerebral small vessel disease (cSVD), are common discoveries in the older population and also in patients with stroke and dementia on cerebral imaging modalities such as magnetic resonance imaging (MRI) and computed tomography (CT) (Wardlaw et al., 2013); on T2‐weighted or flair‐attenuated inversion recovery (FLAIR) on MRI, these brain white matter lesions are usually symmetrically and bilaterally spread in the white matter (Wardlaw et al., 2013). WMH has been reported to increase the risk of stroke, dementia, and death (Burton et al., 2004; Debette & Markus, 2010; Vernooij et al., 2009); furthermore, it has been reported that white matter hyperintensity is a frequent feature of hypertension (de Leeuw et al., 2002; Marcus et al., 2011). A pathological report has shown that patients with WMH undergo demyelination, loss of oligodendrocytes, and axonal damage during the disease cascade (Matsusue et al., 2006). Diffusion tensor imaging studies also echoed the aforementioned hypothesis that axonal damage and impaired white matter integrity occur in WMH (Madden et al., 2012). Although cerebral imaging modalities are widely used in the assessment of WMH in hospitals, undergoing these cerebral imaging is time‐consuming and expensive and some patients may have some contraindications and may not be suitable for undergoing these procedures.

Retinal imaging is now increasingly used in studying diseases related to the brain because of the embryologic, anatomical, and physiological similarities between these two (London, Benhar, & Schwartz, 2013). A surge of evidence has shown that the retina is a reliable, useful, and convenient medium to reflect the changes that occur in the brain (Erskine & Herrera, 2014; London et al., 2013). Previous reports using the OCT have shown that patients with WMH have functional visual deficits and anatomical changes in the retinal structures (Kim et al., 2011; Tak, Sengul, & Bilak, 2018); these studies also showed that patients with WMH undergo loss of retinal ganglion cells and have reduced retinal nerve fiber layer (RNFL) when compared to the healthy controls. Reports from these studies suggested that thinning of the RNFL may be present before the appearance of clinical manifestations and thus may be a manifestation of subclinical brain disease.

With the improvement in the resolution of the retinal imaging modalities, segmentation of the retina has become more convenient and useful in the monitoring the progression of diseases. It has been reported that the RNFL is composed of axons, while the ganglion cell–inner plexiform layer (GCIP) contains the cell bodies and dendrites (Ong et al., 2015); with the retina being postulated as a mirror of the brain, it has been suggested that the GCIP reflects more of the gray matter of the brain, while the RNFL reflect the changes that occur in the cerebral white matter (Mutlu et al., 2017).

In our current study, we conducted an observational study to investigate the association of the retinal sublayer thickness on OCT with focal markers of brain tissue on MRI in participants with WMH using the Fazekas scale.

## 2 METHODS

### 2.1 Study population

The study population consisted of 110 people who participated in cerebral MRI and ophthalmological examination. The study was initiated in January 2016 and ended in December 2017. This observational, cross‐sectional study was approved by the institutional review board for human research at the Second Affiliated and Yuying Hospital of Wenzhou Medical University, China, and written informed consent was obtained from each participant. All subjects were treated in accordance with the tenets of the Declaration of Helsinki.

### 2.2 Collection of clinical data

Data were collected on participants' demographics and laboratory data. Body mass index (BMI) was defined as measured weight (kg) divided by height squared (m^2^). A participant was classified as a smoker if he or she smoked >1 cigarette per day for >1 year; a participant was classified as a drinker if he/she drank at least 100 g of alcohol per day for >1 year. Blood pressure (BP) was measured under standardized conditions. Arterial hypertension was defined as follows: definite if participant was on treatment for hypertension or had a systolic BP ≥140 mmHg or diastolic BP ≥90 mmHg; borderline hypertension if self‐reported hypertension but with no treatment or systolic BP 140–159 mmHg or diastolic BP 90–94 mmHg; or normotensive. Hypercholesterolemia was defined as fasting serum total cholesterol >5.72 mmol/L or participant had started taking statins. All participants underwent Mini‐Mental State Examination (MMSE) and Montreal Cognitive Assessment (MoCA) evaluate the cognitive status before MRI and ophthalmological examination.

The inclusion criteria for WMH patients were as follows: (a) aged from 60 years or older, (b) Chinese Mandarin speaking, (c) sufficient sensorimotor and language competency for cognitive testing, (d) functional independence by a score of 20 on the 20‐point Barthel index and <2 on Lawton's instrumental of daily living scale (IADL), and (e) provided written informed consent. The exclusion criteria for WMH were as follows: (a) history of stroke or transient ischemic attack ascertained by medical records on the electronic health record (Clinical Management System) of the Neurology Department, Second Affiliated Hospital, and Yuying Children's Hospital of Wenzhou Medical University, (b) history of neurological or psychiatric conditions affecting cognitive functions, (c) dementia determined by medical history, (d) evidence of brain tumors, cerebral infarcts larger than 20 mm in diameter, or hydrocephalus on MRI, and (e) subjects with medial temporal lobe atrophy as defined by a rating of >2 rated on coronal images on T1‐weighted brain MRI using Schelten's 5‐point scale to exclude prodromal Alzheimer's disease. Additional criteria for retinal image acquisition included: (f) patients with known retinal disease or disease influencing vessel structure in color retinal images such as diabetic retinopathy, age‐related macular degeneration, central serous chorioretinopathy, postcataract extraction, and retinal pigment epithelial detachment, (g) disturbance of consciousness, and (h) high refractive error (±6.00 D spherical equivalent).

### 2.3 Cerebral structural MRI

All eligible participants underwent MRI scans on an equipment equipped with 3.0 Tesla superconducting magnets (Signa HDxt GE Healthcare; T1‐weighted, T2‐weighted, diffusion‐weighted imaging, fluid‐attenuated inversion recovery [FLAIR]), susceptibility weighted imaging (SWI) axial sequences, and T1‐weighted sagittal sequences. Axial images were angled to be parallel to the anterior commissure–posterior commissure line. Trained and certified radiologists, who were incognito to the participants' clinical condition and retinal imaging findings, assessed the digitized scan data on a personal display workstation at the MRI reading center. When evaluating for WMHs, focal abnormalities were ignored; therefore, if a side or both sides of the brain were focally abnormal, estimates were based on the uninvolved areas. The spin density images (repetition time of 3,000 ms; echo time of 30 s) were used to estimate the overall volume of periventricular and subcortical white matter signal abnormality. Slice thickness was 6 mm, with an interslice gap of 20%.

### 2.4 Visual scoring of WMH burden

The reference standards of the presence of white matter abnormalities have been previously published (Fazekas, Chawluk, Alavi, Hurtig, & Zimmerman, 1987; Smith, Saposnik, et al., 2017). Controls and WMH were rated by two radiologists, who were blinded to their clinical information, based on FLAIR and T2‐W images using Fazekas scale (Fazekas et al., 1987). A total score ranging from 0 to 6 was the sum of periventricular and subcortical Fazekas scores. The *k*‐coefficient for interobserver agreement was 0.912; disagreement was resolved with other specialized radiologists.

**Figure 1:**
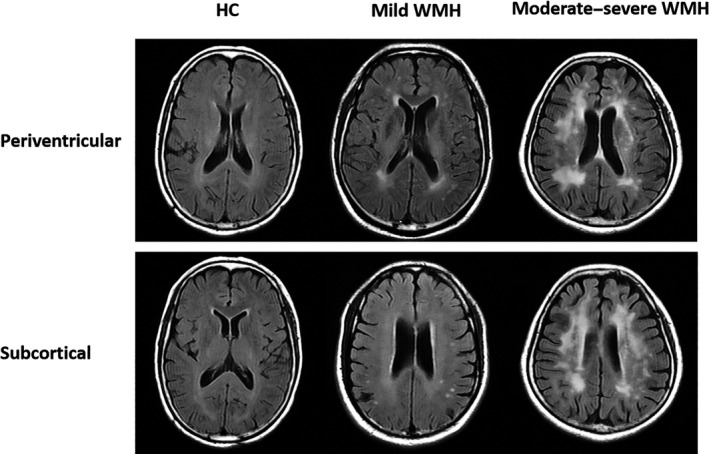
Grading of the white matter hyperintensities using the Fazekas scale for the healthy controls, mild white matter hyperintensity (WMH), and moderate–severe WMH

Participants were divided into three groups by the total Fazekas scores as follows: healthy controls (HC group) without white matter hyperintensity, a Fazekas score of 0; mild WMH (WMH1 group), a Fazekas score of 1–3; and moderate–severe WMH (WMH2 group), a total Fazekas score of 4–6 (Munoz Maniega et al., 2017; Figure 1).

### 2.5 Retinal photography

Photographs of the retina and optic disks were taken of each participant in this study using the fundus camera. Trained ophthalmologists at the Eye Hospital of Wenzhou Medical University, who were masked to the participants' characteristics, evaluated the photographic slides for the presence of abnormalities in the macular and optic disk. Abnormalities of the macular and optic disk were defined as present if any of the following lesions were detected, retinal hemorrhages, soft and hard exudates, macular edema, optic disk swelling, and microaneurysms, and excluded.

The healthy controls, who were without diabetes and well‐controlled hypertension, underwent neurological examinations to rule out neurological diseases.

### 2.6 Spectral‐domain optical coherence tomography procedure and data collection

**Figure 2:**
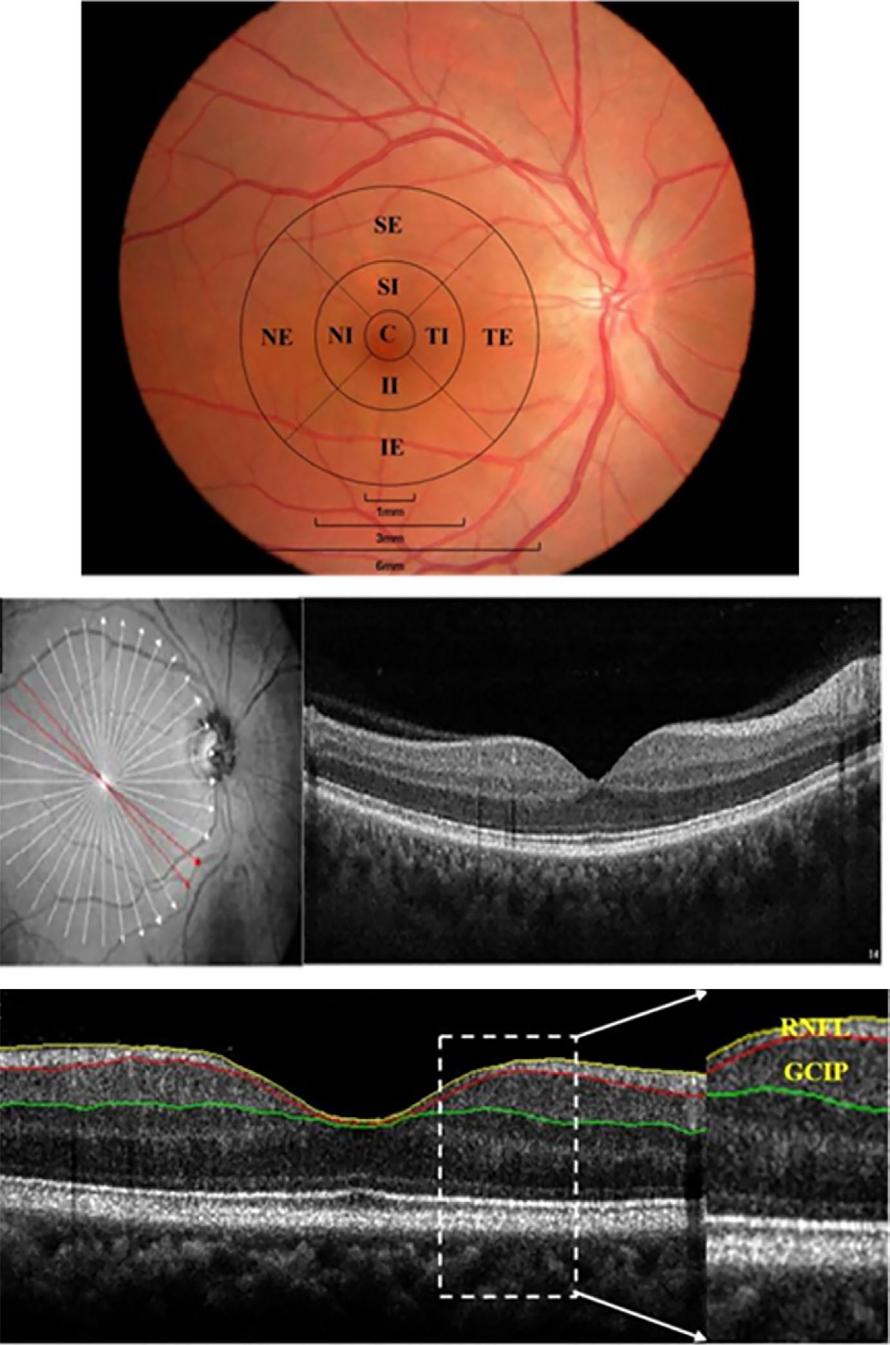
Layering and partitioning of the retina SD‐optical coherence tomography images

The RTVue XR Avanti Spectral Domain OCT system (Optovue, Inc.) was used to image the macula. Imaging of the macula was done by a single, well‐trained examiner. Acquisition of the macula retina was done with a radial scanning mode of 18 lines to generate the 3D thickness map (Figure 2). In every image, a diameter of 6 mm in the area of the macula was taken (Figure 2). A good set of scans, with a signal strength index (SSI) of >40 for each eye, was selected for further analysis. After the acquisition of the image, a custom algorithm was used to segment the retina and measure the intraretinal layer thicknesses as described in our previously published articles (Cheng et al., 2016; Kwapong et al., 2018). Retinal layers were checked for errors and manually segmented using MatLab v.7.10 (Mathworks, Inc.). Bennett's formula was used to correct the changes in axial length (Nowroozizadeh et al., 2014). In our current study, the average thickness of the RNFL and GCIP was measured (Figure 2).

### 2.7 Statistical analysis

All data were expressed as mean ± standard deviation and analyzed using SPSS 23.0 (IBM). Refraction data were converted to spherical equivalents, which were calculated as the spherical dioptric power plus one‐half of the cylindrical dioptric power. In our current study, we used generalized linear models for comparing thickness measures for retinal layers between hypertensive WMH and controls without and with adjustment of participants' demographics (age, hypertension, diabetes, smoking history, and BMI). The intereye correlation of thickness for participants contributing two eyes was accounted for by using generalized estimating equations. Similar analyses were made for comparisons across each subgroup of WMH versus controls using generalized linear models. The thickness calculated from these linear regression models (with or without adjustment for demographic features) was summarized as mean and standard error. Univariate regression analysis and multiple regression analysis were used to determine the risk factors for changes in the RNFL and GCIP layers. *p*‐Values less than .05 were considered to be statistically significant.

## 3 RESULTS

A total of 110 participants were enrolled in this study, of whom eight were excluded because of cerebral acute stroke, seven were excluded because of cataract surgery within the last 6 months, and seven because of a long history (>15 years) of diabetes mellitus. In the end, 88 participants completed the study, comprising of 32 (56 eyes) as the healthy controls (HC group), 28 (53 eyes) patients as the mild WMH group (WMH1 group), and 28 (48 eyes) as the moderate–severe WMH group (WMH2 group). The demographic and clinical characteristics of the enrolled participants are shown in Table 1. The three groups did not differ significantly in gender composition, age, mean arterial pressure (MAP), or ophthalmological findings (*p* > .05, Table 1).

**Table 1:** Comparison of baseline characteristics in hypertensive controls and patients with white matter hyperintensities

	HC (*n* = 32, 56 eyes)	WMH1 (*n* = 28, 53 eyes)	WMH2 (*n* = 28, 48 eyes)	*p*
Male	11	9	11	.68^b^
Age, year	65.12 (6.95)	65.75 (6.26)	65.96 (7.46)	.73^a^
Smoke	6	5	6	.86^b^
Alcohol	4	3	3	.80^b^
Hypercholesterolemia	10	14	15	.60^b^
BMI, kg/m^2^	24.63 (2.19)	24.48 (2.82)	23.92 (3.27)	.62^a^
SBP, mmHg	133.04 (15.93)	134.42 (18.79)	134.61 (12.35)	.93^a^
DBP, mmHg	75.04 (12.77)	75.85 (11.33)	76.57 (9.29)	.90^a^
MAP, mmHg	94.37 (12.26)	95.37 (12.99)	95.91 (9.77)	.90^a^
SE, D	0.87 (1.73)	0.53 (1.87)	0.82 (1.43)	.56^a^
BCVA, Snellen chart	1.17 (0.24)	1.01 (0.21)	1 (0.20)	.69^a^
IOP, mmHg	13.98 (2.87)	13.6 (2.54)	13.54 (2.99)	.72^a^
AL, mm	22.91 (0.7)	23.19 (0.7)	22.95 (0.87)	.17^a^

Note

Values are mean (standard deviation).

Abbreviations: AL, axial length; BCVA, best‐corrected visual acuity; BMI, body mass index; DBP, diastolic blood pressure; HC: healthy controls without white matter; IOP, intraocular pressure; MAP, mean arterial pressure; MWH2: moderate–severe hypertensive white matter hyperintensity; SBP, systolic blood pressure; SE, spherical equivalent; WMH1, mild hypertensive white matter hyperintensity.

^a^ ANOVA.

^b^ Chi‐squared test.

### 3.1 Retinal thickness between healthy controls and hypertensive white matter hyperintensity

When compared to the HC, hypertensive WMH group showed significantly reduced (*p* < .05, Table 2) RNFL and GCIP, respectively. Moderate–severe hypertensive WMH showed significantly reduced (*p* < .05) RNFL and GCIP when compared to HC respectively.

**Table 2:** Comparison of the average retinal sublayer thickness between the hypertensive controls, mild WMH, and moderate–severe WMH participants

	Average thickness	
	RNFL	GCIP
HC	29.01 (4.15)	69.14 (3.81)
WMH	26.55 (3.51)	64.42 (4.21)
Mild WMH	27.25 (3.41)	65.41 (3.82)
Moderate–severe WMH	25.79 (3.49)	63.32 (4.38)
*p* a	<.001	<.001
p*^b^	.017	<.001
*p* ^1^ c	<.001	<.001
*p* ^2^ d	.037	.013
*p* e	.081	<.001
p* f	.633	<.001
*p* ^1^ g	.018	<.001
*p* ^2^ h	.032	.013

Note

Data are presented as mean (standard deviation).

Abbreviations: GCIP, ganglion cell layer and inner plexiform layer; HC, healthy controls; RNFL, retinal nerve fiber layer; WMH, white matter hyperintensity.

a Comparison between healthy controls and hypertensive WMH.

b Comparison between healthy controls and mild hypertensive WMH.

c Comparison between healthy controls and moderate–severe hypertensive WMH.

d Comparison between mild hypertensive WMH and moderate–severe hypertensive WMH.

e Comparison between healthy controls and hypertensive WMH after adjustment for age, smoking, hypertension, diabetes, number of eyes used, and BMI.

f Comparison between healthy controls and mild hypertensive WMH after adjustment for age, smoking, hypertension, diabetes, number of eyes used, and BMI.

g Comparison between healthy controls and moderate–severe hypertensive WMH after adjustment for age, smoking, hypertension, diabetes, number of eyes used, and BMI.

h Comparison between mild hypertensive WMH and moderate–severe hypertensive WMH after adjustment for age, smoking, hypertension, diabetes, number of eyes used, and BMI.

### 3.2 Association between the retinal thickness and Fazekas scores

**Figure 3:**
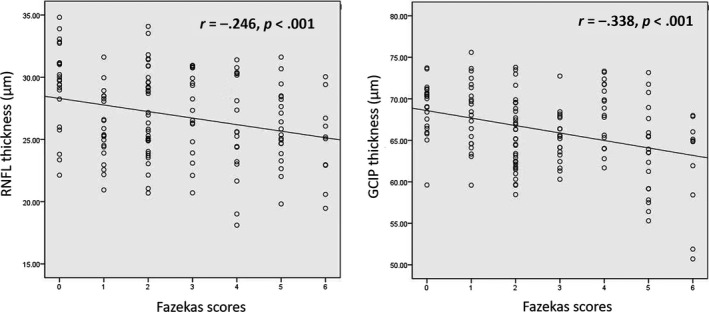
Spearman rank correlation between the retinal thickness and Fazekas score

A significant correlation was found between the RNFL (*ρ* = −.246, *p* < .001; Figure 3) and GCIP (*ρ* = −.338, *p* < .001; Figure 3) of the total participants and the Fazekas score, respectively. Statistical differences were still significant (*p* < .05) when correlations were adjusted for intereye correlation, age, hypertension, smoking, BMI, and diabetes.

### 3.3 Risk factors for thinning of the RNFL and GCIP

Table 3 gives a summary of the risk factors for the thinning of RNFL and GCIP in WMH patients. Variables such as gender, age, BMI, diabetes, hypertension, and smoking history with MRI findings using Fazekas scores were subjected to univariate analysis. In univariate regression analysis, older age and Fazekas scores were significantly associated with the thinning of both the RNFL and GCIP (Table 3). Multivariate linear regression analysis showed that Fazekas scores, BMI, and older age increased the risk factor for thinning of the RNFL, and Fazekas scores, smoking history, and older age increased the risk factor for thinning of the GCIP (Table 4).

**Table 3:** Univariate regression analysis of the risk factors for retinal average thickness

	Regression coefficient	*SE*	Standardized regression coefficient	*t*	*p*
RNFL					
Males	−.219	0.627	−.029	−0.350	.727
Age, year	−.116	0.040	−.235	−2.888	.004
BMI, kg/m^2^	−.248	0.102	−.199	−2.423	.017
Smoke	−.645	0.718	−.075	−0.898	.371
Fazekas scores	−.526	0.154	−.274	−3.406	.001
GCIP					
Males	−.971	0.815	−.099	−1.192	.235
Age, year	−.168	0.052	−.261	−3.233	.002
BMI, kg/m^2^	−.026	0.136	−.016	−0.190	.850
Smoke	−2.335	0.919	−.208	−2.539	.012
Fazekas scores	−.899	0.195	−.359	−4.599	<.001

Note

Univariate regression analysis, Total score: the sum of Fazekas scores for subcortical and periventricular.

Abbreviation: *SE*, standard error.

**Table 4:** Multivariate regression analysis of the risk factors for retinal average thickness

	Regression coefficient	*SE*	Standardized regression coefficient	*t*	*p*
RNFL					
Fazekas scores	−.475	0.150	−.247	−3.159	.002
BMI, kg/m^2^	−.297	0.097	−.238	−3.066	.003
Age, year	−.107	0.039	−.217	−2.761	.007
GCIP					
Fazekas scores	−.797	0.190	−.318	−4.183	<.001
Smoke	−2.354	0.841	−.209	−2.800	.006
Age, year	−.135	0.049	−.209	−2.743	.007

Note

Multivariate linear regression analysis. Total score: the sum of Fazekas scores for subcortical and periventricular.

Abbreviation: *SE*, standard error.

## 4 DISCUSSION

With cerebral atrophy being a typical finding in WMH (Al‐Janabi et al., 2018), we have shown in our present study that thinning of the RNFL and GCIP was independently associated with focal lesions in hypertensive white matter of the brain and deteriorates with the severity of the lesions. Moreover, our study also showed that both damages in the retina (thinning of the sublayers of the retina) and brain (focal lesions) may already be present in the subclinical period, during which there are no apparent clinical symptoms. With the retina being a medium through which the changes in the brain can be viewed in vivo, our current study also suggests that the OCT can be used as a complement tool with the MRI to help in the monitoring of the WMH patients.

Our present study showed that thinning of the RNFL and GCIP was associated with lesions that occur in the cerebral microstructure (i.e., the lesions of the white matter as seen on the MRI), and thinning of these retinal sublayers deteriorates with the severity of the disease/lesions. Accumulating evidence using the DT‐MRI has shown that patients with WMH present with abnormalities in the cerebral microstructure (white and gray matter) throughout the entire cerebral structure (Pelletier et al., 2015; Svard et al., 2017); our present study suggests that thinning of the RNFL and GCIP may echo the subtle changes in the cerebral microstructure which cannot be detected in vivo in patients with WMH.

A novel finding in our study is that thinning of the RNFL and GCIP were associated with severity of the white matter lesions on MRI using the Fazekas scale. Given the link between the retina and the brain, our findings echoes the association between the retina and the brain in WMH as previously reported in the other cerebral small vessel diseases such as Alzheimer's disease (Debette & Markus, 2010; Rhodius‐Meester et al., 2017). Although microstructural changes in the white matter have been noted for its liability to microvascular damage (Connor et al., 2017; Lin, Wang, Lan, & Fan, 2017), our study highlighted the role of neurodegeneration in the white matter damage on MRI images using the Fazekas scale; further studies on the association between the retinal vasculature and the cerebral microstructure are needed to validate this hypothesis and also evaluation of the optic nerve could be investigated. With regard to aging, we observed that increase in age was associated with thinning of the retinal layers and susceptibility to lesions in the white matter which is congruent with previous reports (Harwerth, Wheat, & Rangaswamy, 2008; Mutlu et al., 2017).

After careful evaluation of the images from the MRI, we suggest that specific regions of the susceptible to white matter lesions may have contributed to the thinning of the retinal sublayers analyzed in our current study. Damage to these regions may have caused the damage to the connections involving the visual tract thus causing degeneration of the optic nerve resulting in the changes of these sublayers (Doety, Sandra, & Cornelissen, 2016; Jindahra, Petrie, & Plant, 2009). Visual complaints have been reported to be common in patients with WMH (Allen, Spiegel, Thompson, Pestilli, & Rokers, 2015; Verma, Gupta, & Chaudhari, 2014), and in our current study, we showed that the visual acuity (although not worse in our study) was reduced with the severity in our study. Furthermore, these sublayers have been reported to play a role in visual processing (Smith, Vianna, & Chauhan, 2017), and as such, any damage in these layers may affect the vision of an individual; thus, changes in these layers may translate into the changes that occur in the brain. On the contrary, it may be possible that cell death in the ganglion region of the retina causes anterograde deterioration leading to the changes that occur in the RNFL and may further lead to the white matter lesions seen in the brain which covers the visual tract responsible for vision as reported by Ohno‐Matsui (2011). Although we did not evaluate or give an account on the optic nerve head, our suggestions may need further investigations as the optic nerve is the barrier between the brain and the retina; as such, further studies are needed to validate this hypothesis.

White matter hyperintensity is a radiological finding which may cause loss of axons and neurons in both the retina and the brain. Whether the loss of axons and neurons in the retina and brain occurs simultaneously needs to be further investigated. It has been reported that the GCIP which contains neuronal cell bodies and dendrites and the RNFL which contains axons reflect the gray and white matter of the brain, respectively (Mutlu et al., 2017). However, in our current study, the fact that both the RNFL and GCIP were associated with the severity of the WM lesions using the Fazekas scale makes the previous hypothesis invalid. Longitudinal studies with larger samples are needed to investigate deeper on this.

Our data raise the possibility that asymptomatic participants with hypertensive WMH detected by MRI may benefit from retinal evaluation. However, retinal evaluation was done with custom‐built algorithm which may not be easily translated to clinical practice. Further investigations using a simplified retinal evaluation might increase the practical utility of our data.

There was some limitation in our current study that needs to be addressed. To begin with, the cross‐sectional design of our study limits us to draw conclusions about the cause and effect; longitudinal studies with larger sample sizes are needed to investigate more on our current study. As with most diagnostic tests, patient cooperation is an obligation. Movement from the participant can diminish the quality of the image, and some participants were excluded from the study because of eye movement during OCT examination, and as such, some images were excluded due to poor imaging (SSI < 40); improvement of the eye fixation mode where imaging can be done during eye movement would help make the imaging tool more convenient. Furthermore, we did not evaluate the microstructural integrity of the MRI images; further studies on the microstructural volume of WMH persons may be needed to give an in‐depth meaning.

In conclusion, retinal degeneration in the RNFL and GCIP was independently associated with focal lesions in the white matter of the brain and deteriorates with the severity of the lesions, providing evidence that neurodegeneration is allied to the pathogenesis of WMH. We suggest that imaging and measurement of the retinal sublayers using the OCT may provide evidence on neurodegeneration in WMH.

## CONFLICT OF INTEREST

The authors have no proprietary interest in any materials or methods described in this article.

## DISCLOSURE

The authors have no proprietary interest in any materials or methods described in this article.

## Data Availability

The data that support the findings of this study are available on request from the corresponding author. The data are not publicly available due to privacy or ethical restrictions.
